# Direct-visualization implantation of an atrial leadless pacemaker at tricuspid valve surgery

**DOI:** 10.1016/j.hrcr.2026.07.014

**Published:** 2026-07-21

**Authors:** Pejman Mansouri, Shelief Y. Robbins-Juarez, Francis Hartge, Mark J. Kearns, Ulrika Birgersdotter-Green

**Affiliations:** 1 Division of Cardiovascular Medicine, Department of Medicine; 2 Division of Cardiothoracic Surgery, Department of Surgery, University of California San Diego, San Diego, California

**Keywords:** Atrial leadless pacemaker, Leadless cardiac pacing, Intraoperative pacemaker implantation, Tricuspid valve surgery, Tricuspid regurgitation

**Key Teaching Points**

• This case highlights the first reported intraoperative implantation of an atrial leadless cardiac pacemaker during tricuspid valve surgery and demonstrates that this approach is technically feasible, safe, and associated with stable electrical parameters.
• Surgical implantation of leadless pacemakers under direct visualization offers numerous advantages, including precise device positioning and orientation, avoidance of the need for fluoroscopy or additional large-bore venous access, and avoidance of an additional postoperative procedure for device reimplantation if required.
• Compared with the modest early threshold improvement typically reported after standard transcatheter Aveir atrial implantation, direct-visualization surgical atrial leadless implantation may show a more marked early threshold decline despite an initially high threshold when a substantial current of injury confirms secure fixation.
• In general, leadless atrial pacing offers an attractive alternative to standard transvenous pacemakers for patients who are at high risk of developing or have already developed significant tricuspid regurgitation.

## Introduction

Leadless cardiac pacemakers (LCPs) are an emerging alternative to transvenous pacing systems, offering the potential to reduce lead-related complications.^1^ In particular, the avoidance of transtricuspid leads is desirable in patients with tricuspid valve (TV) disease or previous valve interventions. Chronic pacemaker leads crossing the TV can precipitate significant tricuspid regurgitation (TR). Studies have reported pacemaker lead–induced TR in approximately 10%–40% of patients owing to mechanical leaflet interference or perforation.^2^, ^3^, ^4^ With the growing number of TV surgeries and transcatheter interventions, there is a rising interest in pacing strategies that preserve TV integrity.^2^^,^^4^ Among alternative strategies, leadless pacemakers are especially attractive because they eliminate any hardware traversing the TV and thus minimize the risk of valvular damage or dysfunction. In addition, leadless systems obviate the subcutaneous pocket and implanted leads, significantly reducing infection risk and avoiding interference with prosthetic or repaired valves.^5^^,^^6^

Until recently, leadless pacing has been largely limited to the right ventricle (RV) with single-chamber ventricular devices. Dual-chamber leadless systems (eg, Abbott Aveir DR) have now been developed, enabling atrial pacing in an entirely leadless configuration.^7^ However, clinical experience with atrial leadless pacemakers remains very limited. To the best of our knowledge, direct-visualization endocardial implantation of an atrial leadless pacemaker during open-heart surgery has not been previously reported; previous intraoperative leadless pacing cases have primarily involved RV devices implanted during tricuspid interventions.^8^^,^^9^ Here, we report a novel case of intraoperative endocardial implantation of an atrial leadless pacemaker during TV repair.

## Case presentation

A 40-year-old woman with a history of syncope and intermittent atrioventricular (AV) block underwent dual-chamber pacemaker implantation 7 years earlier. The original pacemaker indication was syncope in the setting of documented intermittent AV block, rather than presumed vasovagal syncope alone. She had no recurrence of syncope after pacemaker implantation. Over time, she developed severe symptomatic TR attributed to the RV pacing lead crossing the TV (pacing-induced TR). Pacemaker interrogation demonstrated minimal pacing requirements, with 0% ventricular pacing and approximately 10% atrial pacing, consistent with preserved native AV conduction at the time of evaluation and no ongoing ventricular pacing requirement. The device was programmed DDD with a lower rate of 60 beats/min (bpm), upper tracking rate of 140 bpm, and programmed AV delays of 280/250 ms. Before the procedure, atrial pacing was performed through the existing transvenous pacemaker in AAI mode up to 150 bpm, demonstrating intact 1:1 AV conduction. Given her worsening TR and preserved native conduction, the patient was referred for surgical correction of the TV with concurrent removal of the transvenous pacemaker system. The operative plan included TV repair and implantation of an atrial leadless pacemaker.

The procedure was performed via a median sternotomy. Intraoperatively, the old pacemaker generator and leads (right atrial and RV) were completely extracted. The TV was found to have severe regurgitation with leaflet tethering. A tricuspid annuloplasty ring (28 mm) was placed, and the septal leaflet was augmented with a patch, successfully restoring valve competence. After completing the valve repair, the atrial leadless pacemaker was implanted through the same oblique right atriotomy used for TV exposure and repair; no separate atriotomy was required. The atrial LCP (Abbott Aveir AR2) was positioned at the base of the right atrial appendage under direct visualization. Because this was a reoperative procedure, the atrial tissue was generally thicker, with some burden of epicardial adhesions, and the tissue at the selected implant site was considered adequate for fixation. The device’s screw-in helix was actively fixated into the atrial myocardium by directly rotating the device while stabilizing the right atrial appendage; printed device markings provided a visual reference, and fixation was achieved with approximately 1.5–2 rotations (Figure 1; Supplemental Video 1). No specialized delivery sheath or fluoroscopy was required for deployment. Secure fixation was confirmed with gentle traction, and there was no evidence of full-thickness helix penetration or microperforation.

**Figure 1:**
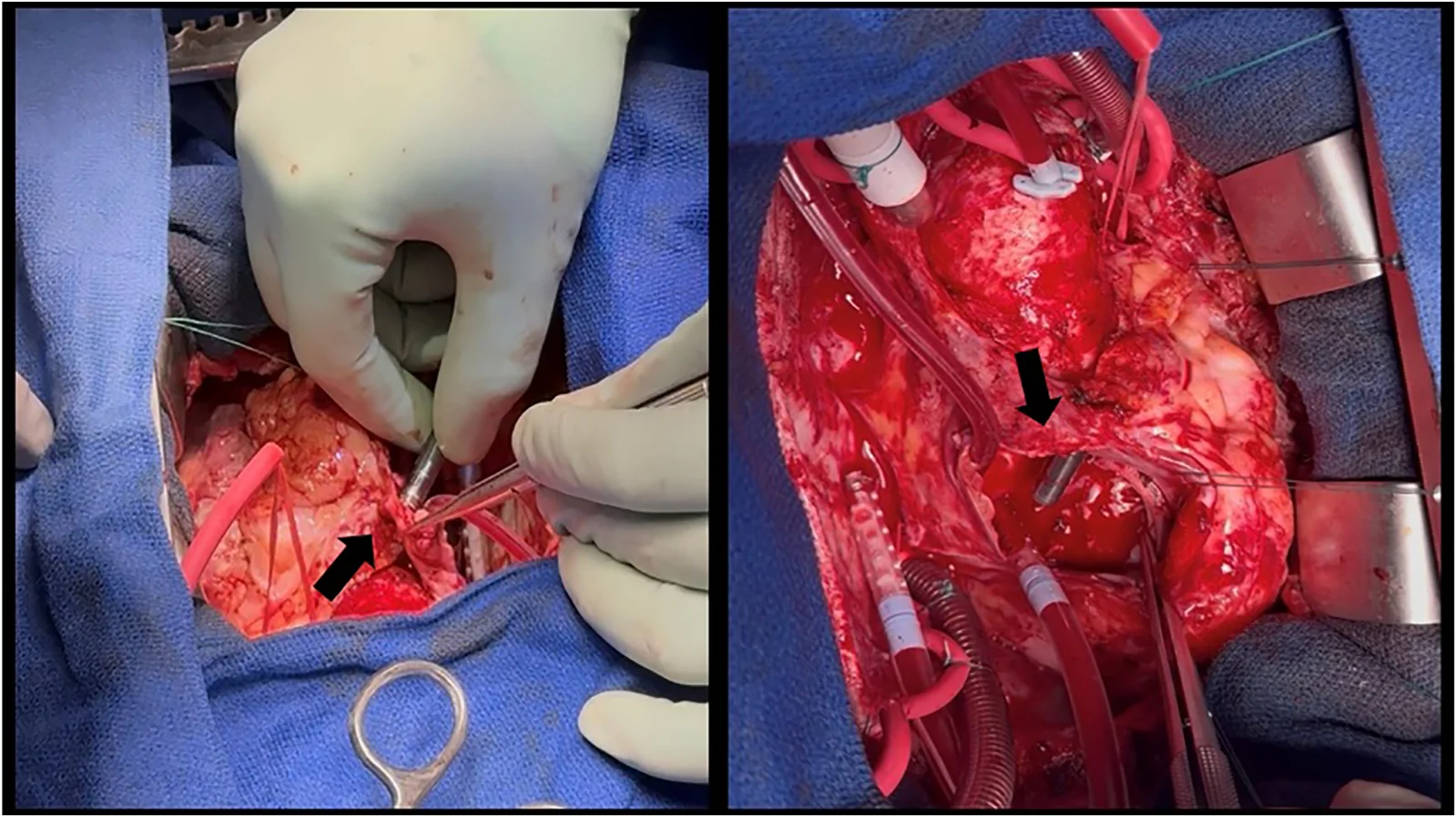
Direct-visualization intraoperative implantation of an atrial leadless pacemaker (indicated by *black arrows*) during tricuspid valve repair.

Intraoperative testing of the leadless atrial pacemaker showed an initial atrial capture threshold of 3.75 V at 0.4 ms pulse width, with a P-wave sensing amplitude of 0.25 mV. A significant current of injury was noted at the implant site (Figure 2), indicating good tissue contact and anticipating improvement in electrical parameters. The device’s pacing impedance measured approximately 310 Ω. By the end of the case, and on repeat testing at chest closure, the threshold had already begun to decrease. The pacemaker was programmed in AAI mode with a lower rate limit of 60 bpm. A postoperative chest radiograph confirmed the LCP in proper position within the right atrium, with no transvenous leads present (Figure 3).

**Figure 2:**
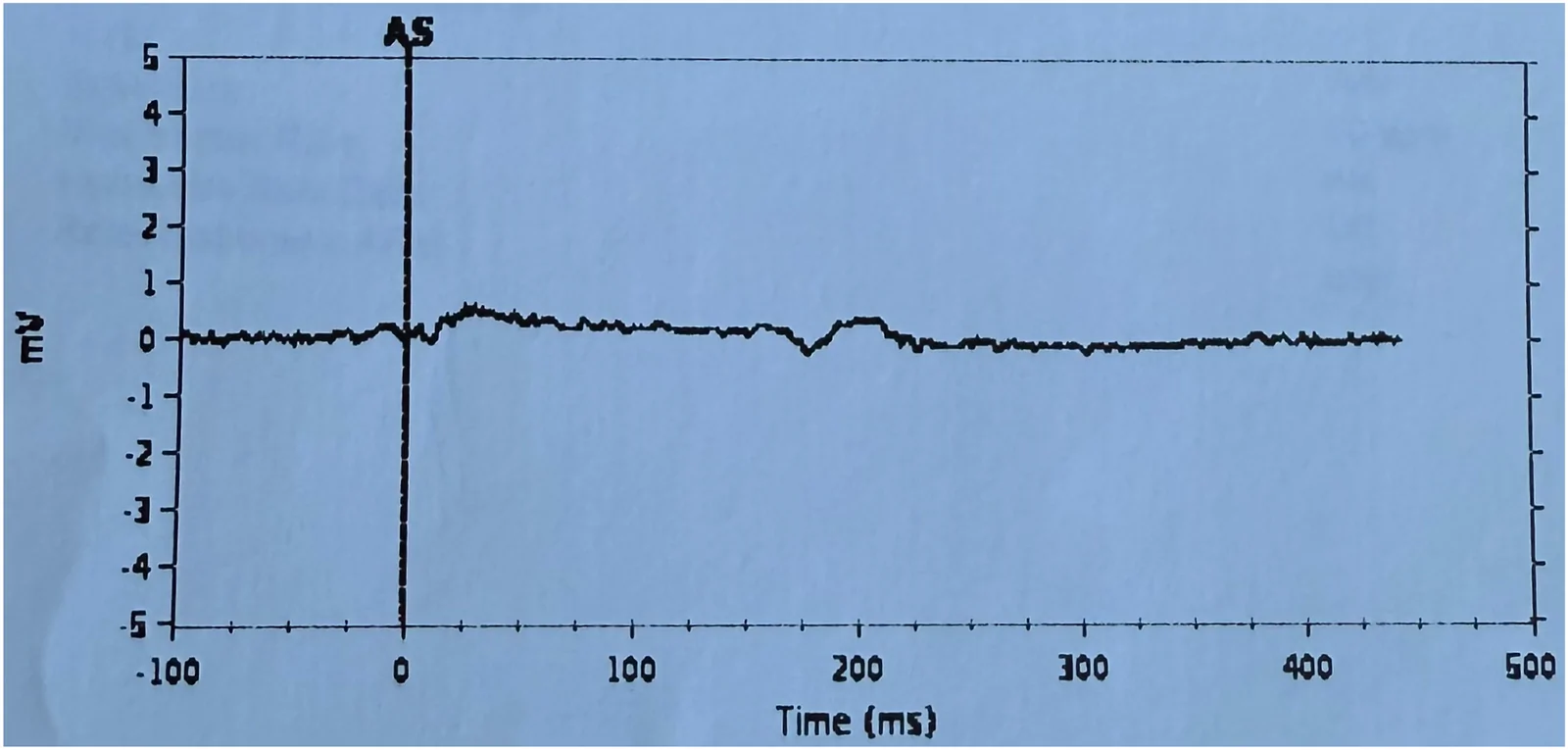
Intracardiac electrogram demonstrating current of injury at the implantation site.

**Figure 3:**
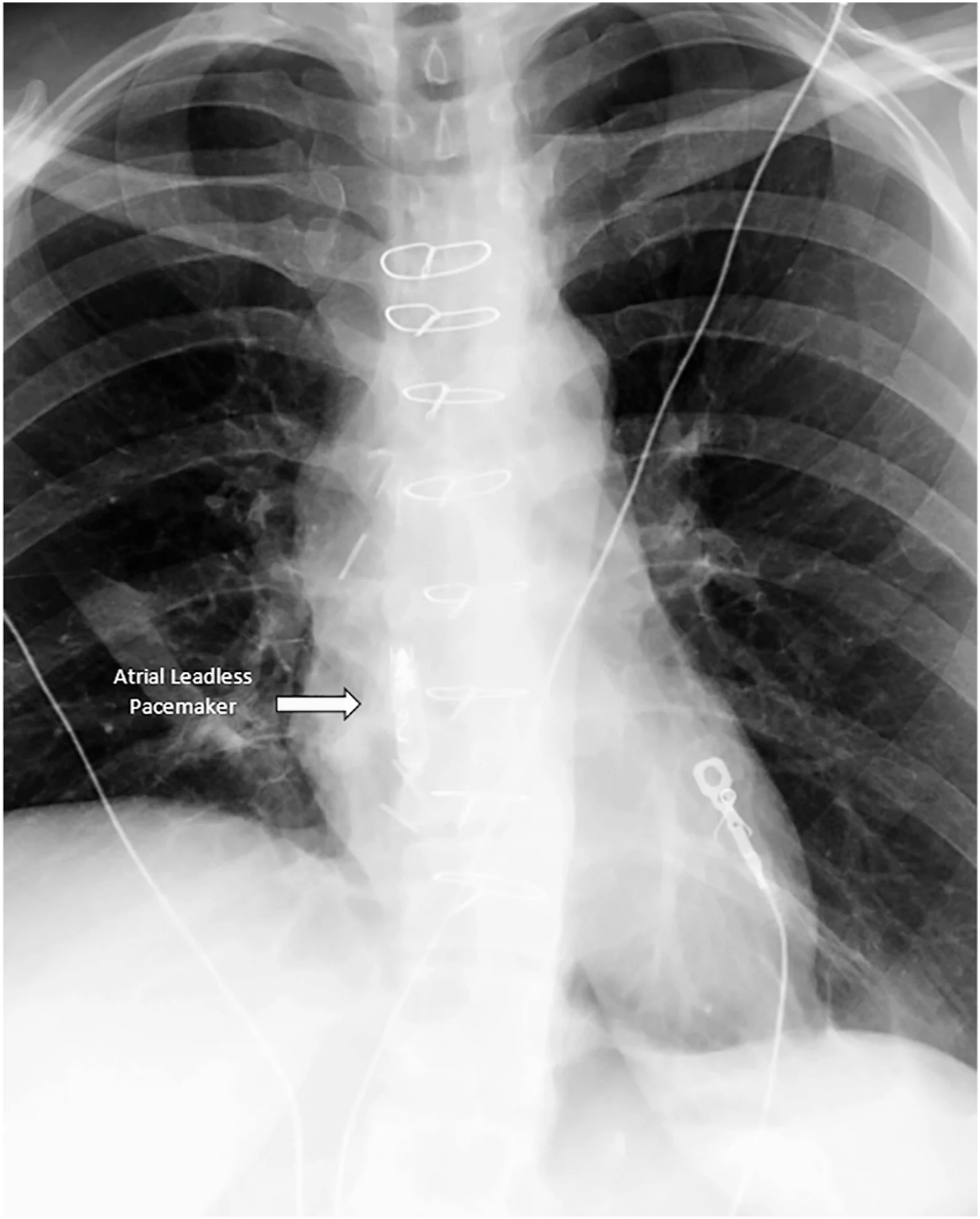
Postoperative chest radiograph confirming atrial leadless pacemaker position.

The patient’s recovery was uncomplicated. She remained in sinus rhythm with intact AV conduction throughout her hospital course. The day after surgery, telemetry showed 1:1 native AV conduction without any ventricular pauses; no separate ventricular pacing system was required. By discharge, the atrial leadless pacemaker demonstrated markedly improved electrical performance: the atrial capture threshold was 0.5 V at 0.4 ms, sensed P-wave amplitude 0.5 mV, and impedance ∼300 Ω. The LCP was programmed to an output of 2.75 V at 0.4 ms, projecting an estimated battery longevity of ∼16 years at this setting. An implantable loop recorder was placed before discharge for long-term rhythm monitoring, given the patient’s history of heart block and to document her AV conduction status over time. At the 2-week follow-up, the patient was clinically well. Device interrogation confirmed stable parameters and continued normal sinus rhythm with appropriate atrial pacing, whereas implantable loop recorder monitoring demonstrated intact AV conduction.

## Discussion

To the best of our knowledge, this report describes the first case of intraoperative implantation of an atrial LCP. The favorable clinical outcome demonstrates the technical feasibility and short-term safety of surgically implanting an atrial LCP and highlights a novel pacing strategy for selected patients undergoing TV or other right-sided cardiac surgeries.

With an aging population and expanding use of cardiac implantable electronic devices (CIEDs), the prevalence of CIED-associated and CIED-induced TR has increased substantially.^10^ For patients who develop severe symptomatic TR despite optimal medical therapy, procedural intervention of the TV is often indicated, as well as consideration of lead extraction with possible device replacement. Although there are several percutaneous options to address TR by removing or jailing leads, including transvenous lead extraction and transcatheter TV replacement, surgical intervention remains a cornerstone for those who are not considered to be at high surgical risk.^11^^,^^12^ For pacing-dependent patients undergoing surgical intervention, it would have been previously indicated to place an epicardial lead, which carries a nontrivial risk of bleeding and infection.^5^

In the case presented, the patient was a 40-year-old woman with a low Society of Thoracic Surgeons risk score and was appropriately recommended for surgical management. Given her intact AV conduction system and absence of any ventricular pacing requirement, an atrial-only pacing strategy was pursued. The available management options were discussed with the patient, and she strongly preferred to maintain pacing support and elected to proceed with atrial leadless pacemaker implantation at the time of surgery. This approach also avoided new transtricuspid hardware and a separate postoperative implantation procedure. Leadless atrial pacemakers became clinically available after approval of the dual-chamber leadless system (Aveir, Abbott Medical) in 2023 and have shown favorable early safety and performance outcomes.^7^ Compared with traditional transvenous systems, atrial LCPs mitigate the standard risks of transvenous leads, including lead infection, TV damage, and risks associated with creating a subcutaneous generator pocket, including infection and bleeding. These advantages are a particularly relevant consideration in younger patients.^6^

Although the number of leadless pacemaker implantations has markedly increased over the past decade, there are currently scarce data regarding leadless pacemaker implantation during cardiac surgery. Several case reports describe surgically implanted RV LCPs. 1 case similarly presented a leadless device placed during surgical TV repair, and another case described pacemaker implantation during surgical removal of an RV mass.^8^^,^^9^ Eltsov et al^13^ reported the first concomitant epicardial implantation of a leadless pacemaker on the right atrial appendage during Maze IV surgery in a young woman with atrial fibrillation and sinus node dysfunction. In our case, an atrial LCP was successfully implanted intraoperatively with immediate confirmation of fixation and appropriate device parameters. There were no immediate or postoperative complications, and she was doing very well at her postdischarge follow-up with excellent pacing threshold and sensing amplitude. These cases highlight the numerous advantages of surgical implantation under direct visualization, including precise device position, confirmation of stable fixation, avoidance of fluoroscopy, and avoidance of an additional postoperative procedure for device implantation.^5^^,^^6^

In conclusion, this case demonstrates that surgically implanted atrial leadless pacing is feasible and can be safely performed in conjunction with TV surgery. This strategy represents a promising option for carefully selected patients and may mitigate many of the limitations associated with transvenous and epicardial systems. Direct-visualization intraoperative deployment offers important technical and clinical advantages, particularly in redo sternotomy or complex valvular cases, by enabling precise positioning and secure fixation in a controlled operative field, avoiding fluoroscopy and specialized delivery systems, and potentially sparing the patient a separate postoperative implantation procedure. Further clinical experience and long-term follow-up are needed to better define patient selection, durability, and outcomes associated with this evolving approach.

## Disclosures

Dr Birgersdotter-Green has received grant support from Abbott Laboratories and honoraria from Medtronic, Abbott, and Boston Scientific. The remaining authors declare no conflicts of interest.
