# Supplementary material for: Direct-visualization implantation of an atrial leadless pacemaker at tricuspid valve surgery
Source: HeartRhythm Case Rep. 2026 Jul 21;12(10):1236–9. doi: 10.1016/j.hrcr.2026.07.014 (PMC13687968; doi:10.1016/j.hrcr.2026.07.014)
Supplement: Supplementary file 1 [file mmc1.docx]

Supplementary Video Legend

Video 1. Direct-visualization intraoperative implantation of an atrial leadless pacemaker during tricuspid valve repair.
